# In vitro analysis of various mouthwashes: cytotoxic, apoptotic, genotoxic and antibacterial effects

**DOI:** 10.1007/s00784-025-06261-0

**Published:** 2025-03-15

**Authors:** Dilşah Çoğulu, Aslı Aşık, Sunde Yılmaz Süslüer, Ceren Yücel Er, Aslı Topaloğlu, Ataç Uzel, Cumhur Gündüz

**Affiliations:** 1 Department of Pediatric Dentistry, Faculty of Dentistry, Ege University, İzmir, Turkey; 2 Department of Pediatric Dentistry, Faculty of Dentistry, İzmir Tınaztepe University, İzmir, Turkey; 3 Department of Medical Biology, Faculty of Medicine, Ege University, İzmir, Turkey; 4 Pediatric Dentist, Menemen Oral and Dental Health Center, İzmir, Turkey; 5 Department of Pediatric Dentistry, Faculty of Dentistry, İstanbul University-Cerrahpaşa, İstanbul, Turkey; 6 Division of Basic and Industrial Microbiology Section, Department of Biology, Faculty of Science, Ege University, Izmir, Turkey

**Keywords:** Biocompability, Cytotoxicity, Chlorhexidine gluconate, Cetylpyridinium chloride, Mouthwashes, Antibacterial

## Abstract

**Objective:**

This study aims to investigate the cytotoxic, apoptotic, and genotoxic effects of eleven mouthwashes, which are hypothesized to pose potential toxic risks to oral mucosal tissues, through in-vitro experiments using human gingival fibroblast(hGF) cell cultures and to compare the antibacterial efficacy of these mouthwashes.

**Materials and methods:**

Healthy hGF cell lines were derived from individuals under ethical standards. The cytotoxic effects of the mouthwashes (Colgate 2in1,Colgate Optic White, Colgate Plax, Curasept, Elmex, Kloroben, Listerine Cool Mint, Listerine Zero, Meridol, Oral-B Pro Expert, Sensodyne Pronamel) were assessed in real-time using the xCELLigence system, which monitored cellular activity at 5-minute intervals over 72 h. Apoptotic effects of the IC50 concentrations of the mouthwashes on hGF cells were evaluated using Annexin V and Caspase-3 assays. Genotoxic effects at IC50 concentrations were analyzed through the Alexa Fluor^®^ 488 Mouse anti-H2AX assay. The antibacterial effect of mouthwashes on *Streptococcus mutans* and *Lactobacillus rhamnosus* was evaluated by modified microdilution method.

**Results:**

According to the analysis of the IC50 values, Meridol was found to be the most cytotoxic mouthwash, while Listerine Zero was the least cytotoxic. The mouthwashes can be ranked in order of their cytotoxicity as follows: Meridol(0.011) > Elmex(0.029) > Colgate 2in1(0.187) > Colgate Plax(0.478) > Colgate Optic White (0.534) > Sensodyne Pronamel(0.577) > Oral-B Pro Expert(0.638) > Kloroben(0.766) > Curasept(1.872) > Listerine Cool Mint(2.334) > Listerine Zero(7.267)(*p* < 0.05). The Annexin V assay showed no major apoptotic impacts on human gingival fibroblast cell line at the IC50 values of the mouthwashes, except for Colgate Plax, Kloroben, and Oral B Pro Expert. The IC50 values of the evaluated mouthwashes did not show a significant apoptotic effect on the hGF cell line as evaluated by Caspase-3 assay and no significant genotoxic effect was observed as evaluated by H2AX assay(*p* > 0.05). Colgate Plax showed the most effective antibacterial effect on *Streptococcus mutans* and *Lactobacillus rhamnosus*(*p* < 0.05).

**Conclusion:**

Mouthwash formulations showed varying cytotoxic effects on hGF and different antibacterial effectiveness. Essential oil-containing mouthwashes may be preferable to those with chlorhexidine or cetylpyridinium chloride, as they demonstrate less cytotoxicity, are more biocompatible, and have antibacterial efficacy.

**Clinical relevance:**

The low cytotoxicity and potent antibacterial properties of essential oils render them a more safer choice for inclusion in mouthwash compositions.

## Introduction

The regular removal of microbial dental plaque is critical for the prevention of gingival diseases and dental caries. Effective management of dental plaque is recognized as the most fundamental strategy for preventing periodontal diseases and dental caries. This involves promoting optimal oral hygiene practices, including mechanical plaque removal through brushing and flossing, as well as the use of antimicrobial agents in mouthwashes. Consistent and thorough plaque removal reduces the microbial load in the oral cavity, thereby minimizing the risk of inflammation and subsequent periodontal tissue destruction, as well as inhibiting the progression of carious lesions. Adhering to these practices is essential for maintaining oral health and preventing the onset of related diseases. However, in instances where mechanical plaque control proves insufficient due to individual limitations or contextual factors, the use of chemical agents targeting pathogenic microorganisms becomes indispensable. This imperative has spurred ongoing research initiatives focused on the development of tailored antimicrobial agents designed for individual use. Researchers are exploring various compounds, including essential oils, natural extracts, and synthetic agents, to identify those with optimal efficacy against oral pathogens while minimizing adverse effects on oral tissues. Advances in formulations and delivery mechanisms, such as nanoencapsulation and sustained-release systems, are also being investigated to enhance the bioavailability and effectiveness of these antimicrobial agents. This research is crucial in addressing the diverse needs of individuals, particularly those with specific oral health conditions or heightened susceptibility to microbial infections. Ultimately, the goal is to create personalized oral care products that provide targeted antimicrobial action, improve adherence to oral hygiene practices, and contribute to the prevention of oral diseases. Currently, a variety of chemical compounds with established antibacterial properties are incorporated into mouthwash formulations for the prevention and management of plaque-related oral diseases [1].

The utilization of antimicrobial agents in mouthwashes has gained increased significance in light of the COVID-19 pandemic, as these agents not only play a crucial role in the prevention of periodontal diseases and dental caries but also contribute to the mitigation of infectious disease transmission. Furthermore, they facilitate the execution of aerosol-free dental procedures, reducing the risk of pathogen spread in clinical settings. This dual functionality underscores the growing importance of mouthwashes containing antimicrobial compounds in both oral healthcare and infection control especially during the pandemic [2].

The primary active ingredients commonly utilized in this context are antimicrobial agents, including chlorhexidine, cetylpyridinium chloride, essential oils, chlorine dioxide, triclosan, amine fluoride, stannous fluoride, hydrogen peroxide, and hyaluronic acid. These compounds are incorporated into mouthwash formulations due to their demonstrated efficacy in inhibiting the growth of pathogenic microorganisms and contributing to the prevention of oral diseases [3].

Mouthwash formulations display considerable variability in composition, yet there are several essential attributes expected of an ideal mouthwash. An optimal mouthwash should consist of non-toxic, non-allergenic, and non-irritating ingredients. Furthermore, it should demonstrate efficacy in controlling dental plaque, thereby playing a key role in mitigating the development of plaque-induced periodontal diseases and dental caries. These characteristics are critical in ensuring both the safety and therapeutic effectiveness of mouthwash products in oral health care [4]. Mouthwashes, as oral care products intended for regular use, come into direct contact with the soft tissues and gingiva within the oral cavity. Despite their widespread use, limited studies have investigated the biocompatibility of mouthwashes with varying compositions, particularly their effects on human gingival fibroblast cells [5–8].

The objective of this study is to assess the cytotoxicity, apoptotic potential, and genotoxicity of various mouthwashes, which are hypothesized to have toxic effects on oral mucosal tissues, through in vitro cell culture experiments using human gingival fibroblasts and also to compare the antibacterial properties of these mouthwashes against *Streptococcus mutans* and *Lactobacillus rhamnosus*.

## Materials and methods

The study was conducted in accordance with the principles outlined in the Declaration of Helsinki and received approval from the Ethics Committee of Ege University, Faculty of Medicine (protocol number:14 − 5.1/7).

### Isolation of human gingival fibroblast (hGF) cells

Human gingival fibroblast cells utilized in this study were derived from two healthy individuals undergoing tooth extractions for orthodontic reasons at Ege University, Faculty of Dentistry, Department of Periodontology. Gingival tissue samples were collected under sterile conditions with the application of local anesthesia. The specimens were then transferred to the Department of Medical Biology at Ege University, Faculty of Medicine, where the tissues were enzymatically digested using Collagenase Type II (Biochrom AG, Berlin, Germany) to create a suspension culture. Dulbecco’s Modified Eagle’s Medium (DMEM; Biochrom AG, Berlin, Germany), supplemented with 10% Fetal Bovine Serum (FBS), 1% L-glutamine, and penicillin (10,000 U)/streptomycin (10,000 µg/ml), was employed as the culture medium. Cells were incubated at 37 °C in a humidified incubator (Heraeus^®^, Berlin, Germany) with 95% humidity and 5% CO_2_ until they reached adequate growth. Cell proliferation, passage, and follow-up were monitored using an inverted microscope, with each cell line subcultured in separate flasks. Third-passage cells were subsequently used for cytotoxicity, apoptosis, and genotoxicity assays.

### Preparation of different mouthwashes

The mouthwashes used in the present study were commercially available, ready-to-use formulations, with their specific ingredients listed in Table 1. In the initial experimental phase, 1 mL of each mouthwash; Colgate Plax, Colgate Optic White, Sensodyne Pronamel, Oral B Pro Expert, Kloroben, Curasept, Listerine Cool Mint, and Listerine Zero was diluted in 9 mL of cell culture medium to achieve a 10% concentration. Additionally, 10 µL of each mouthwash—Meridol, Elmex, and Colgate 2in1—was diluted in 9.99 mL of cell culture medium to create a 0.1% concentration. To ensure homogenous mixtures, a vortex mixer was employed. The resulting diluted solutions were then filtered through a 0.22 μm pore-size filter, producing sterile DMEM culture media containing mouthwash concentrations of 10% and 0.1%, respectively.

**Table 1:** Composition of mouthwashes used in the present study

Mouthwashes	Manufacturer	Ingredients
Colgate 2in1	Colgate-Palmolive, New York, USA	Water, Sorbitol, Hydrated Silica, Glycerin, Sodium Lauryl Sulfate, Aroma, Tetrasodium Pyrophosphate, Sodium Saccharin, Cocamidopropyl Betaine, 0. 24% Sodium Fluoride, Cellulose Gum, Xanthan Gum, Limonene, CI 77,891
Colgate Optic White	Colgate-Palmolive, New York, USA	Water, Glycerin, Propylene Glycol, Sorbitol, Tetrapotassium Pyrophosphate Polysorbate 20 Tetrasodium Pyrophosphate Zinc Citrate, PVM/MA Copolymer, Aroma, Benzyl Alcohol, 225 ppm Sodium Fluoride, Sodium Saccharin, Cl 42,051.
Colgate Plax	Colgate-Palmolive, New York, USA	Water, Glycerin, Propylene Glycol, Sorbitol, Poloxamer 407, Aroma, Cetylpyridinium Chloride, 0.05% Sodium Fluoride, Methylparaben, Menthol, Sodium Saccharin, Propylparaben, CI42051.
Curasept	Curaden AG, Kriens, Switzerland	Water, Xylitol, Propylene Glycol, PEG-40 Hydrogenated Castor Oil, Ascorbic Acid, Chlorhexidine Digluconate, Aroma, Sodium Fluoride, Poloxamer 407, Sodium Benzoate, Sodium Metabisulfite, Sodium Citrate, CI 42,090.
Elmex	Gaba International, Therwil, Switzerland	Water, PEG-40 Hydrogenated Castor Oil, Olaflur, Aroma, Potassium Acesulfame, 250 ppm Sodium Fluoride, Polyaminopropyl Biguanide, Hydrochloric Acid.
Kloroben	Drogsan, Istanbul, Turkey	0.15% Benzidamine Hydrochloride, 0.12% Chlorhexidine Gluconate, Sorbitol, Propylene Glycol, Aroma, Mint Essence, Patent V Blue, Quinoline Yellow
Listerine Cool Mint	Johnson&Johnson, New Jersey, USA	Water, Alcohol, Sorbitol, Poloxamer 407, Benzoic Acid, Sodium Saccharin, Eucalyptol, Aroma, Methyl Salicylate, Thymol, Menthol, Sodium Benzoate, Cl 42,053.
Listerine Zero	Johnson&Johnson, New Jersey, USA	Water, Propylene Glycol, Sorbitol, Poloxamer 407, Sodium Lauryl Sulfate, Benzoic Acid, Sodium Saccharin, Eucalyptol, Aroma, Methyl Salicylate, Thymol, Menthol, Sodium Benzoate, 220 ppm Sodium Fluoride, Sucralose, Cl 42,053.
Meridol	Gaba International, Therwil, Switzerland	Water, xylitol, PVP, PEG-40 hydrogenated Castor Oil, Olaflur, Aroma, 250 ppm Stannous Fluoride, Sodium Saccharin, CI 42,051.
Oral B Pro Expert	Protecter&Gamble, Ohio, USA	Water, Glycerin, Polysorbate 20, Aroma, Methylparaben, Cetylpyridinium Chloride, 0.05% Sodium Fluoride, Sodium Saccharin, Sodium Benzoate, Probilparaben, Cl 42,051, Cl47005.
Sensodyne Pronamel	GlaxoSmithKline, London, UK	Water, Glycerin, Sorbitol, Poloxamer 338, PEG-60 Hydrogenated Castor Oil, VP/VA Copolymer, Potassium Nitrate, Sodium Benzoate, Cellulose Gum, Aroma, 450 ppm Sodium Fluoride, Methylparaben, Propylparaben, Cetylpyridinium Chloride, Sodium Saccharin, Xanthan Gum, Disodium Phosphate, Sodium Phosphate, Cl 42,090.

### Determination of cytotoxic effects of mouthwashes on gingival fibroblast cell line by xCELLigence real-time cell analyzer (RTCA) system

The IC50 value, defined as the concentration that inhibits biological or biochemical activity in 50% of the cell population, was determined for each mouthwash over up to 72 h using the real-time cell analysis (RTCA-xCELLigence) system. This system comprises four primary components: the RTCA Analyzer, the RTCA SP Station, a computer equipped with RTCA software, and single-use 96 E-Plates, which are embedded with gold microelectrodes at the bottom of each well. During measurement, an approximate voltage of 20 mV was applied to the electrodes. Initially, 100 µL of Dulbecco’s Modified Eagle’s Medium (DMEM) was added to each well of the 96-well xCELLigence plate, and the baseline measurement was recorded after placing the plate in the incubator. Subsequently, 100 µL of a cell-medium mixture containing 3 × 10⁵ gingival fibroblast cells per mL was added to each well, and the cells were allowed to adhere to the bottom of the wells for 24 h, with readings taken every 15 min during this period.

Following cell attachment, 100 µL of the medium was withdrawn from each well and replaced with mouthwash solutions at various concentrations: Colgate Plax, Colgate Optic White, Sensodyne Pronamel, Oral B Pro Expert, Kloroben, Curasept, Listerine Cool Mint, and Listerine Zero were diluted in DMEM to final concentrations of 1/10, 1/20, 1/40, 1/80, 1/160, and 1/320, while Meridol, Elmex, and Colgate 2in1 were diluted to concentrations of 1/1000, 1/2000, 1/4000, 1/8000, 1/16000, and 1/32000. Cytotoxicity was monitored over 72 h using the RTCA-xCELLigence system. The IC50 values were calculated by fitting the cytotoxicity data to a sigmoidal dose-response curve using the equation [Y = bottom + (top - bottom) / (1 + 10(Log IC50-X).

### Determination of apoptotic effects of mouthwashes on gingival fibroblast cell line by annexin v and caspase-3 tests

Each well of a 6-well culture plate was seeded with 0.5 × 10⁶ gingival fibroblast cells suspended in 1 mL of culture medium. The cells were incubated under conditions of 95% humidity and 5% CO_2_ for 24 h to allow for adequate attachment to the culture plate surface. After this incubation period, 1 mL of the existing medium was removed from each well and replaced with 3 mL of fresh medium containing the IC50 concentrations of the various mouthwashes. The cells were then exposed to the mouthwash-containing medium for an additional 72 h.

At the end of the 72-hour incubation, the media were aspirated, and 500 µL of trypsin-EDTA was added to each well to facilitate the detachment of the adherent cells. After a 5-minute incubation with trypsin, 1 mL of medium supplemented with 10% fetal bovine serum (FBS) was added to neutralize the trypsin activity. Subsequently, 1.5 mL of the cell suspension from each well was transferred into Eppendorf tubes and centrifuged at 2,500 rpm for 5 min. The supernatant was discarded, and the resulting cell pellets were resuspended in 1 mL of phosphate-buffered saline (PBS), and centrifuged again for 5 min to ensure thorough washing of the cells.

#### For annexin V test

In each experimental tube, 100 µL of binding buffer, 1 µL of Annexin V, and 1 µL of propidium iodide were added. The samples were subsequently incubated at room temperature in the absence of light for 5 min. Following this incubation period, the levels of apoptosis within the cell populations were assessed at the 72-hour time point using a BD Accuri™ C6 Flow Cytometer (Biosciences, USA). The cells were classified into four distinct categories: viable cells, early apoptotic cells, late apoptotic cells, and necrotic cells.

#### For caspase-3 test

After washing with PBS, the cells were resuspended in BD Cytofix/Cytoperm™ solution at a concentration of 1 million cells per 0.5 mL. The suspension was incubated on ice for 20 min, after which the BD Cytofix/Cytoperm™ solution was carefully removed from the cell pellet. Subsequently, the cells were washed twice with BD Perm/Wash™ buffer at room temperature. The assessment of apoptosis within the cell populations was conducted at the 72-hour time point using a BD Accuri™ C6 Flow Cytometer (Biosciences, USA). The cell populations were classified into two distinct groups: apoptotic cells and non-apoptotic cells.

### Determination of genotoxic effects of mouthwashes on gingival fibroblast cell line by H2AX test

The genotoxic effects of the IC50 doses of mouthwashes were assessed utilizing the Alexa Fluor^®^ 488 Mouse anti-H2AX assay method. Following a wash with PBS, the cells were fixed in 3.7% formaldehyde in 100 µL of fresh PBS and incubated for 10 min at room temperature. Subsequently, the fixative was removed, and the cells were permeabilized by incubation in 100 µL of -20 °C 90% methanol for 5 min. After the permeabilization buffer was discarded, the wells were washed with 1000 µL of PBS, and the supernatant was removed. The cells were then incubated with 100 µL of Triton X-100 for 5 min at room temperature, followed by centrifugation at 2500 rpm for 5 min, after which the supernatant was discarded.

A diluted pH2AX antibody (25 µL) was added to each well and incubated for 30 min. For the dilution, 2.5 µL of pH2AX antibody was mixed with 22 µL of PBS and centrifuged to eliminate any unbound antibody. Following this incubation, the cells were treated with 100 µL of 0.05% Tween, centrifuged again, and the supernatant was removed. The cells were then resuspended in 50 µL of PBS, and the resulting genotoxicity within the cells was evaluated using a BD Accuri™ C6 Flow Cytometer (Biosciences, USA) at the 72-hour time point.

### Antibacterial activity of mouthwashes

In this experiment, the modified microdilution method was utilized to evaluate the antibacterial effects of various mouthwashes on *Streptococcus mutans* and *Lactobacillus rhamnosus*. *L. rhamnosus* ATCC 7469 and *S. mutans* ATCC 25,175 were cultured in MRS Broth (Merck, Darmstadt, Germany) and tryptic soy broth (Oxoid, Basingstoke, UK), respectively, to activate the organisms and incubated in 5 mL liquid media at 37 °C for 24–48 h. The microbial cell count was adjusted to 0.5 McFarland standard in saline solution. Antimicrobial activity experiments were conducted using a modified microdilution method in 96-well U-shaped microplates. All trials were performed in triplicate. Positive controls utilized sterile growth medium and the test microorganism, monitoring microbial growth. Negative controls consisted solely of the growth medium, examining for any contamination. The results were evaluated based on the formation of a red color after adding 10 µL of 2,3,5-Triphenyl-tetrazolium chloride (TTC) to the wells at the 24th hour. The presence of red color in the microdilution method signifies active metabolism and growth of the microorganisms, while the absence of red color suggests inhibition or death of the organisms.

### Statistical analysis

The experimental procedures were replicated using gingival fibroblast cells derived from two distinct human donors, and the average results from these tests were calculated. Data analyses were conducted using the IBM SPSS Statistics software (Version 25.0, Armonk, NY, USA). A sigmoidal dose-response analysis was employed to statistically evaluate the toxicological properties of the mouthwashes. Furthermore, statistical assessments of the data obtained from the apoptosis and genotoxicity assays were performed utilizing the chi-square test and estimated relative risk analysis. A significance level of 0.05 was established to determine statistical significance.

## Results

Based on the IC50 values of the mouthwashes determined through the xCELLigence method, the cytotoxic effects on hGF cell lines were ranked as follows: Meridol (0.011) exhibited the highest cytotoxicity, followed by Elmex (0.029) > Colgate 2 in 1 (0.187) > Colgate Plax (0.478) > Colgate Optic White (0.534) > Sensodyne Pronamel (0.577) > Oral B Pro Expert (0.638) > Kloroben (0.766) > Curasept (1.872) > Listerine Cool Mint (2.334) > Listerine Zero (7.267) (*p < 0.05*) (Table 2).

**Table 2:** IC50 values of mouthwashes

Mouthwashes	IC50 Values %
**Colgate 2in1**	0.187
**Colgate Optic White**	0.534
**Colgate Plax**	0.478
**Curasept**	1.872
**Elmex**	0.029
**Kloroben**	0.766
**Listerine Cool Mint**	2.334
**Listerine Zero**	7.267
**Meridol**	0.011
**Oral B Pro Expert**	0.638
**Sensodyne Pronamel**	0.577

When evaluated using the Annexin V assay, no significant apoptotic effects were observed in the hGF cell line at the IC50 doses of the mouthwashes, except Colgate Plax, Kloroben, and Oral B Pro Expert (*p* > 0.05). Colgate Plax mouthwash significantly induced apoptosis in the hGF cell line at a rate of 3.36-fold (odds ratio [OR] = 3.36, 95% confidence interval [CI] = 1.37–8.26, *p* = 0.008). Similarly, Kloroben mouthwash resulted in a significant 3.93-fold increase in apoptosis (relative risk [R] = 3.93, 95% CI = 1.62–9.40, *p* = 0.003). Oral B Pro Expert mouthwash demonstrated the most pronounced effect, inducing apoptosis in the hGF cell line at a rate of 7.05-fold (*R* = 7.05, 95% CI = 2.98–16.69, *p* = 0.000) (Table 3).

**Table 3:** Comparison of apoptotic effects of mouthwashes by Annexin V

Mouthwashes	ALIVE (%)	APOPTOSIS (%)	OR	95% C.I.	*p*
**Control**	92.79	7.22	1.000	-	-
**Colgate 2in1**	98.80	13.17	0.77	0.04–15.66	0.864
**Colgate Optic White**	86.84	13.17	1.95	0.75–5.06	0.170
**Colgate Plax**	79.27	20.73	3.36	1.37–8.26	0.008*
**Curasept**	91.09	8.92	1.26	0.45–3.50	0.659
**Elmex**	95.04	4.96	0.67	0.21–2.18	0.507
**Kloroben**	76.62	23.39	3.93	1.62–9.54	0.003*
**Listerine Cool Mint**	95.76	4.24	0.57	0.17–1.95	0.371
**Listerine Zero**	99.32	0.69	0.09	0.01–1.07	0.057
**Meridol**	94.72	5.28	0.72	0.23–2.28	0.573
**Oral-B Pro Expert**	64.59	35.42	7.05	2.98–16.69	0.000*
**Sensodyne Pronamel**	87.05	12.96	1.91	0.74–4.98	0.183

*: *p* < 0,05

The study revealed that the IC50 doses of the mouthwashes assessed did not demonstrate a significant apoptotic effect on the hGF cell line, as determined by the Caspase 3 assay (*p* > 0.05) (Table 4).

**Table 4:** Comparison of apoptotic effects of mouthwashes by caspase 3

Mouthwashes	INACTIVE CAS3(%)	ACTIVE CAS3 (%)	OR	95% C.I.	*p*
**Control**	99.02	0.98	1.000	-	-
**Colgate 2 in 1**	99.25	0.76	0.77	0.04–15.66	0.864
**Colgate Optic White**	99.12	0.88	0.90	0.05–16.17	0.941
**Colgate Plax**	97.48	2.52	2.61	0.25–27.39	0.423
**Curasept**	99.03	0.97	0.99	0.06–16.62	0.994
**Elmex**	96.45	3.55	3.72	0.39–35.43	0.253
**Kloroben**	98.90	1.10	1.12	0.07–17.35	0.933
**Listerine Cool Mint**	99.71	0.29	0.29	0.01–18.89	0.563
**Listerine Zero**	99.24	0.76	0.77	0.04–15.68	0.867
**Meridol**	99.09	0.92	0.93	0.05–16.34	0.962
**Oral B Pro Expert**	98.55	1.45	1.49	0.11–19.58	0.763
**Sensodyne Pronamel**	98.29	1.71	1.76	0.14–21.39	0.658

Furthermore, no significant genotoxic effects were observed at the IC50 concentrations of the mouthwash formulations on the hGF cell line, as evaluated using the H2AX assay (*p* > 0.05) (Table 5).

**Table 5:** Comparison of genotoxic effects of mouthwashes by H2AX

Mouthwashes	H2AX NEG (%)	H2AX POZ (%)	OR	95% C.I.	*p*
**Control**	97.44	2.57	1.000	-	-
**Colgate 2 in 1**	98.71	1.29	0.83	0.02–31.85	0.0919
**Colgate Optic White**	97.62	2.39	0.72	0.02–32.03	0.864
**Colgate Plax**	98.89	1.12	1.60	0.07–36.88	0.769
**Curasept**	99.01	0.99	0.81	0.02–31.85	0.911
**Elmex**	96.22	3.79	5.38	0.37–79.15	0.220
**Kloroben**	97.11	2.90	3.17	0.19–53.62	0.424
**Listerine Cool Mint**	98.06	1.94	3.04	0.18–52.16	0.443
**Listerine Zero**	99.27	0.73	1.06	0.04–32.65	0.972
**Meridol**	97.93	2.08	0.84	0.02–31.86	0.927
**Oral B Pro Expert**	99.68	0.33	0.36	0.00-42.33	0.673
**Sensodyne Pronamel**	99.22	0.78	1.43	0.06–35.32	0.828

The antibacterial activity was evaluated using the modified microdilution method, and the results are detailed in Table 6. The presence of a red color indicates the activity of the microorganism, which is coded (+) in the Table 6. Conversely, the absence of a red color signifies the death of the microorganism, as indicated (-) in the Table 6.

The results obtained from evaluating the antibacterial efficacy of 11 different mouthwashes using the test are as follows; Colgate Plax, Sensodyne Pronamel and Kloroben were found more effective mouthwashes against *S. mutans.* The order of antibacterial effect against *S.mutans* is as follows from highest to lowest: Colgate Plax = Sensodyne Pronamel = Kloroben > Colgate2in1 = Meridol = Oral-BPro-Expert = Curasept = Elmex > Listerine Zero > Colgate Optic White > Listerine Cool Mint. Colgate Plax was the most effective mouthwash against *L. rhamnosus*. The order of antibacterial effect against *L.rhamnosus* is as follows from highest to lowest: Colgate Plax > Colgate 2in1 = Meridol = Oral-B Pro-Expert = Sensodyne Pronamel > Kloroben = Elmex > Listerine Zero > Curasept > Listerine Cool Mint = Colgate Optic White.

**Table 6:** Comparison of antibacterial effects of mouthwashes by modified microdilution method

		Dilution rates of mouthwashes								
Mouthwashes	Microorganisms	1/2	1/4	1/8	1/16	1/32	1/64	1/128	1/256	1/512
**Colgate 2in1**	*Streptococcus mutans* ATCC 25,175	-	-	-	-	-	-	-	-	+
	*Lactobacillus rhamnosus* ATCC 7469	-	-	-	-	-	-	+	+	+
**Colgate Optic White**	*Streptococcus mutans* ATCC 25,175	-	-	-	+	+	+	+	+	+
	*Lactobacillus rhamnosus* ATCC 7469	-	+	+	+	+	+	+	+	+
**Colgate Plax**	*Streptococcus mutans* ATCC 25,175	-	-	-	-	-	-	-	-	-
	*Lactobacillus rhamnosus* ATCC 7469	-	-	-	-	-	-	-	+	+
**Curasept**	*Streptococcus mutans* ATCC 25,175	-	-	-	-	-	-	-	-	+
	*Lactobacillus rhamnosus* ATCC 7469	-	-	+	+	+	+	+	+	+
**Elmex**	*Streptococcus mutans* ATCC 25,175	-	-	-	-	-	-	-	-	+
	*Lactobacillus rhamnosus* ATCC 7469	-	-	-	-	-	+	+	+	+
**Kloroben**	*Streptococcus mutans* ATCC 25,175	-	-	-	-	-	-	-	-	-
	*Lactobacillus rhamnosus* ATCC 7469	-	-	-	-	-	+	+	+	+
**Listerine Cool Mint**	*Streptococcus mutans* ATCC 25,175	-	+	+	+	+	+	+	+	+
	*Lactobacillus rhamnosus* ATCC 7469	-	+	+	+	+	+	+	+	+
**Listerine Zero**	*Streptococcus mutans* ATCC 25,175	-	-	-	-	-	+	+	+	+
	*Lactobacillus rhamnosus* ATCC 7469	-	-	-	-	+	+	+	+	+
**Meridol**	*Streptococcus mutans* ATCC 25,175	-	-	-	-	-	-	-	-	+
	*Lactobacillus rhamnosus* ATCC 7469	-	-	-	-	-	-	+	+	+
**Oral B Pro Expert**	*Streptococcus mutans* ATCC 25,175	-	-	-	-	-	-	-	-	+
	*Lactobacillus rhamnosus* ATCC 7469	-	-	-	-	-	-	+	+	+
**Sensodyne Pronamel**	*Streptococcus mutans* ATCC 25,175	-	-	-	-	-	-	-	-	-
	*Lactobacillus rhamnosus* ATCC 7469	-	-	-	-	-	-	+	+	+

## Discussion

Mouthwashes commonly contain various active compounds, among which chlorhexidine, a phenyl guanidine derivative and cationic detergent, is one of the most widely utilized. Mouthwash compounds target oral bacterial flora and impact human cells, with the gingiva being particularly susceptible. The present study evaluated the biocompatibility and antibacterial activity of various over-the-counter mouthwashes on human gingival fibroblast (hGF) cells. Eleven mouthwash products were assessed, including. Curasept and Kloroben were found to contain chlorhexidine, Colgate Plax, Oral B Pro Expert, and Sensodyne Pronamel, all containing the active ingredient cetylpyridinium chloride, whereas Colgate 2 in 1, Colgate Optic White, Colgate Plax, Elmex, Oral B Pro Expert, and Sensodyne Pronamel included sodium fluoride as an active ingredient. Notably, Meridol contained stannous fluoride, a distinct fluoride compound. Elmex and Meridol differ further by containing olaflur. Essential oil-based formulations, such as Listerine Cool Mint and Listerine Zero, include active ingredients like thymol, eucalyptol, and menthol.

Chlorhexidine acts as a broad-spectrum antiseptic, exhibiting potent activity against both gram-positive and gram-negative bacteria as well as certain lipophilic viruses. During the pandemic, the emphasis on oral hygiene has intensified, as the oral cavity is recognized as a potential reservoir for respiratory pathogens. The use of antiviral mouthwashes could serve as an adjunctive measure in public health strategies aimed at controlling the spread of COVID-19, particularly in settings where social distancing is challenging. Furthermore, the ability of mouthwashes to reduce viral load may have implications for infection control in clinical environments, thereby enhancing the safety of dental procedures [9–11]. In clinical applications as a mouthwash, chlorhexidine is generally administered at concentrations between 0.12% and 0.2%. However, extended use of concentrations above 0.2% has been linked to adverse effects, including altered taste perception, desquamation of the oral mucosa, and staining of the teeth and tongue. These potential side effects warrant caution regarding long-term chlorhexidine use, especially at higher concentrations [3, 12].

Cetylpyridinium chloride, frequently employed as an active ingredient in mouthwashes, is classified as a cationic surfactant with broad-spectrum antimicrobial properties, particularly effective against oral bacteria [13, 14]. The prolonged use, especially at elevated concentrations, has been linked to adverse effects such as dental calculus formation, oral cavity burning sensations, and desquamation of the oral mucosa, underscoring the need for controlled, limited use to minimize these outcomes [13–17].

Mouthwashes formulated with essential oils commonly include a blend of thymol, eucalyptol, menthol, and methyl salicylate dissolved in an alcohol-based solution. These essential oils possess antimicrobial and anti-inflammatory properties, establishing them as a favorable alternative to chlorhexidine for dental plaque management. This multifaceted mechanism contributes to the efficacy of essential oils in reducing bacterial colonization and promoting oral health [3, 18, 19].

Since the 1940s, fluoride compounds have been extensively employed in dentistry for their dual role in caries prevention and inhibition of oral bacteria growth. Their antibacterial effect is largely attributed to their ability to diminish bacterial acid production, a key factor in caries development [3, 17].

Alcohol is a prevalent constituent in numerous mouthwash formulations, serving primarily as a preservative and solvent to improve formulation stability and facilitate the dissolution of active ingredients. Although conclusive evidence linking alcohol-containing mouthwashes to carcinogenesis remains inconclusive, growing concerns regarding potential health risks have led to an increased preference for alcohol-free alternatives [3, 20].

Among the tested products, Listerine Zero demonstrated the least cytotoxic effect, followed by Listerine Cool Mint. The variation in cytotoxicity is likely attributable to the alcohol content in Listerine Cool Mint, while Listerine Zero contains propylene glycol as an alternative to alcohol. Studies indicate that alcohol-containing mouthwashes may exert toxic effects via protein denaturation, with some research even suggesting a potential link to oral carcinogenesis [21].

In the study by Köhidai et al., the cytotoxicity of eight commercial mouthwashes on human gingival epithelial progenitor (HGEPp) cells was evaluated over 24 and 48 h using xCELLigence analysis and Annexin-V apoptosis assay. Essential oil-based mouthwashes, such as Listerine Cool Mint and Listerine Fluoride Plus, exhibited lower cytotoxic effects compared to other formulations. For instance, Listerine Cool Mint’s IC50 values at 24 and 48 h were as low as 0.001, contrasting with an IC50 of 2.33 in human gingival fibroblast (hGF) cells at 72 h in this study, suggesting increased tolerance over prolonged exposure. Moreover, Annexin V results indicated that essential oil-based mouthwashes did not trigger apoptosis, reinforcing their lower cytotoxicity profile [22]. Our findings were in accordance with the results of Köhidai et al.’s study.

Bayraktar et al. used the MTT assay to assess the impact of a 0.2% chlorhexidine solution on the viability and cytotoxicity of human gingival fibroblasts (hGF). Findings indicated that 0.2% chlorhexidine significantly decreased hGF cell viability, demonstrating cytotoxic effects even after short exposure times of 30 s and 2 min [23]. Our findings similarly indicate that chlorhexidine-containing mouthwashes exhibit higher cytotoxicity levels compared to those formulated solely with essential oils.

Müller et al. investigated the cytotoxicity of 12 commercial mouthwashes on human gingival fibroblasts using a formazan formation assay, determining the lethal concentration (LC50) after a 2-minute exposure. Results showed LC50 values over 20% for Listerine, categorizing it as moderately toxic, whereas Meridol and Elmex had LC50 values below 20%, indicating high cytotoxicity [24]. These findings align with the present study, which also identified Meridol and Elmex as exhibiting the highest cytotoxicity levels.

Oliveira et al. examined the effects of five mouthwash formulations (Cepacol Traditional, Colgate Plax, Listerine Cool Mint, Oral B Complete, and Sensodyne) on gingival fibroblast cell viability (FMM-1 cells) using the MTT assay at 24 h post-application. The study concluded that all tested products significantly decreased cell viability, supporting the findings observed in our current study [25]. The findings of our study are consistent with existing literature, reinforcing previously reported observations.

The current literature on mouthwash-induced genotoxicity is limited [26–31]. In the present study, no statistically significant genotoxic effects were observed in human gingival fibroblast cells, although DNA damage varied by formulation. Elmex, Meridol, and Kloroben showed the highest levels of DNA damage, while Listerine Zero, Oral B Pro Expert, and Sensodyne Pronamel exhibited minimal DNA damage. Previous research by Khan et al. indicated possible genotoxic effects of chlorhexidine-containing mouthwash on buccal epithelial cells, suggesting that the 0.12% chlorhexidine content in Kloroben warrants further investigation into chlorhexidine’s potential DNA-damaging effects [26]. Additional studies are essential to elucidate the long-term effects and underlying mechanisms associated with mouthwash-induced cytotoxicity and genotoxicity in gingival cells. Expanding research in this area could provide a more comprehensive understanding of formulation-specific impacts on oral cell viability and DNA integrity, ultimately guiding safer product development and usage recommendations.

In the present study, both the biocompatibility and the antibacterial efficacy of various mouthwashes against pathogenic microorganisms within the oral flora were assessed. The current study assessed the antibacterial effects of mouthwashes on the *S. mutans* species, which is a major contributor to dental caries. Additionally, the study investigated the impact of mouthwashes on beneficial bacteria, such as *L. rhamnosus*, that have a positive influence on oral health. Beneficial bacteria like *L. rhamnosus* inhibit the growth of cariogenic bacteria such as *S. mutans*. One desirable characteristic of an ideal mouthwash is the ability to exhibit antibacterial activity against harmful bacterias while allowing the presence of beneficial bacterias. Findings indicated that the mouthwash containing cetylpyridinium chloride, sodium fluoride, and menthol (Colgate Plax) exhibited the most potent antibacterial effects against *Streptococcus mutans* and *Lactobacillus rhamnosus*. The present study revealed that the evaluated mouthwash products exerted an antimicrobial influence on both the beneficial and detrimental bacteria comprising the oral microbiome. Mouthwashes with cetylpyridinium chloride and chlorhexidine demonstrated significantly stronger antibacterial activity compared to those containing solely essential oils. Supporting these findings, Olejnik et al. evaluated the antibacterial efficacy of six mouthwashes (Colgate Plax, Listerine Professional, Oral-B Pro-Expert, Mint Perfekt Sensitiv, Elmex, and Smile 3D Protection), concluding that Colgate Plax was the most effective against *Streptococcus mutans* [32]. Similarly, in another study, Demirel et al. investigated the antibacterial effects of six commercially available mouthwashes (Colgate Total, Colgate Plax, Colgate Proargin, Oral-B Pro-Expert, Listerine, and Oderol), finding that mouthwashes containing chlorhexidine and cetylpyridinium chloride were more effective, consistent with the results of the present study [33].

While these findings provide valuable contributions to the current literature, the in vitro study design has inherent limitations in replicating the complex intraoral environment. Protective factors such as salivary flow rate, buffering capacity, mucosal barriers, creatinine levels, and native oral flora significantly modulate the oral cavity’s resilience to potentially harmful agents. Moreover, this study focused solely on healthy human gingival fibroblasts, underscoring the need for future research that includes gingival fibroblasts from periodontally diseased tissues. Such studies would yield a more comprehensive understanding of mouthwash effects across varying periodontal conditions.

In conclusion, this study highlights the critical impact of mouthwash composition on both cytotoxicity and antibacterial efficacy, underscoring the nuanced relationship between biocompatibility and microbial control in oral health care. Specifically, variations in active ingredients (such as cetylpyridinium chloride, chlorhexidine, and essential oils) play a significant role in determining the balance between antibacterial potency and cytotoxic risk to oral tissues. These findings emphasize the need for a judicious approach in prescribing mouthwashes, where preference should be given to formulations that achieve robust antibacterial action while minimizing cytotoxic effects on soft tissues. Clinicians are therefore advised to recommend mouthwashes that maximize biocompatibility, aiming to support oral health without compromising tissue integrity.

## Conclusion

Mouthwash formulations exhibit distinct cytotoxic profiles on human gingival fibroblast cell lines, with antibacterial efficacy closely tied to their unique compositions. Notably, essential oil-based mouthwashes emerge as promising alternatives, displaying significantly reduced cytotoxicity compared to formulations with chlorhexidine or cetylpyridinium chloride. This reduced cytotoxicity highlights the potential of essential oils to balance potent antimicrobial effects with enhanced cellular compatibility, positioning them as attractive candidates in the development of safer, effective oral hygiene products.

## Data Availability

The entirety of the data analyzed in this study has been included in the published article. Upon making a reasonable request, the corresponding author will provide access to the raw data.
